# Review and Outcome of Prolonged Cardiopulmonary Resuscitation

**DOI:** 10.1155/2016/7384649

**Published:** 2016-01-14

**Authors:** Houssein Youness, Tarek Al Halabi, Hussein Hussein, Ahmed Awab, Kellie Jones, Jean Keddissi

**Affiliations:** ^1^University of Oklahoma, 920 Stanton L Young Boulevard, WP 1310, Oklahoma City, OK 73104, USA; ^2^Lebanese American University, Lebanon

## Abstract

The maximal duration of cardiopulmonary resuscitation (CPR) is unknown. We report a case of prolonged CPR. We have then reviewed all published cases with CPR duration equal to or more than 20 minutes. The objective was to determine the survival rate, the neurological outcome, and the characteristics of the survivors.* Measurements and Main Results*. The CPR data for 82 patients was reviewed. The median duration of CPR was 75 minutes. Patients mean age was 43 ± 21 years with no significant comorbidities. The main causes of the cardiac arrests were myocardial infarction (29%), hypothermia (21%), and pulmonary emboli (12%). 74% of the arrests were witnessed, with a mean latency to CPR of 2 ± 6 minutes and good quality chest compression provided in 96% of the cases. Adjunct therapy included extracorporeal membrane oxygenation (18%), thrombolysis (15.8%), and rewarming for hypothermia (19.5%). 83% were alive at 1 year, with full neurological recovery reported in 63 patients.* Conclusion*. Patients undergoing prolonged CPR can survive with good outcome. Young age, myocardial infarction, and potentially reversible causes of cardiac arrest such as hypothermia and pulmonary emboli predict a favorable result, especially when the arrest is witnessed and followed by prompt and good resuscitative efforts.

## 1. Introduction 

Cardiopulmonary resuscitation (CPR) using closed-chest cardiac massage technique was first used in 1960 by Kouwenhoven in 17 patients with cardiac asystole and 3 patients with ventricular fibrillation, with a successful resuscitation in 14 patients (70%) [1]. There are currently no firm guidelines regarding the duration of such resuscitation [2]. In one large study, the overall median duration of resuscitation for in-hospital cardiac arrest was 17 minutes with an interquartile range of 10–26 min [3]. For out-of-hospital arrest, the National Association of Emergency Medical Services Physicians suggest that resuscitative efforts could be terminated in patients who do not respond to at least 20 minutes of advanced life support care [4]. The current overall survival rate to hospital discharge is 17% for in-hospital cardiac arrest and 5% for out-of-hospital cardiac arrest [5, 6]. Despite this overall poor prognosis, multiple cases of successful prolonged resuscitation with neurologically intact survival have been reported.

In this paper we describe a case of prolonged resuscitation with successful outcome. Since there is no randomized trials that have evaluated the duration of resuscitation and the bulk of information regarding the duration of resuscitation in various conditions such as hypothermia relies on case series and expert opinion, we reviewed all reported cases of prolonged resuscitation in the English literature. Our goal was to determine the success rate and neurological outcome of these patients and to define the characteristics of patients who might benefit from such heroic measures.

## 2. Case Report

A 76-year-old African American man was admitted to the orthopedics service with a right midtibial fracture. He has a past medical history of diabetes, anemia, obstructive sleep apnea, hypertension, and osteoarthritis. He was taken to the operating room (OR) for open reduction and internal fixation under general anesthesia with endotracheal intubation and sciatic nerve block. Pulse oximetry, end tidal CO_2_ (ETCO_2_), bispectral index (BIS), and arterial line blood pressure were continuously monitored. Patient received intravenous midazolam and fentanyl and was paralyzed with cisatracurium.

During the procedure, he was in sinus rhythm with heart rate of 120 per minute, blood pressure was 136/71, and temperature was 36.2°C, with FiO_2_ of 0.6 with O_2_ saturation of 98%, ETCO_2_ of 37 mmHg, and BIS ranging from 39 to 48 during the entire procedure.

After three and half hours of general anesthesia, the tourniquet was released, and immediately the blood pressure dropped to 50/30 mmHg, the heart rate decreased to 20 beats per minute, O_2_ saturation dropped to 80, and the ETCO_2_ dropped to 7 mmHg. The patient became pulseless and CPR was immediately started. The initial rhythm was pulseless electrical activity (PEA). Epinephrine and vasopressin were given intravenously. Acute massive pulmonary embolism was suspected. Aspiration from left internal jugular central line was done for possible air embolism. Emergent transesophageal ultrasound was performed and showed a dilated right ventricle, with poor systolic function, and hyperdynamic left ventricle. Heparin bolus of 10,000 units was administered. Systemic thrombolytic therapy was contraindicated with the open leg wound. Despite resuscitation for 20 minutes, the patient remained in PEA. The catheterization lab was activated to attempt percutaneous embolectomy. CPR was continued en route with continuous ETCO_2_ monitoring which was maintained above 20 mmHg. Multiple providers switched every 2-3 minutes to maintain good quality chest compression, as measured by the end tidal CO_2_. Upon arriving in the catheterization laboratory the patient had return of spontaneous circulation (ROSC) to normal sinus rhythm. Total CPR duration was 86 min during which the rhythm remained PEA. No cardioversion was performed. Medications given during CPR included 5 mg of epinephrine followed by a drip at 50 micrograms/min, 40 units of vasopressin, 2 mg of atropine, 100 meq (100 mmol) of bicarbonate, and 2 gm (13.6 mmol) of calcium chloride for low ionized calcium level (1.09 mmol/L, normal in our lab is greater than 1.12 mmol/L). Bicarbonate was given due to the concern for severe acidosis in the context of prolonged PEA (pH down to 6.9 during the code). BIS was continuously monitored and remained above 80 during the entire resuscitation.

Pulmonary angiogram revealed multiple bilateral distal pulmonary emboli. No saddle or proximal pulmonary artery emboli were seen. A retrievable inferior vena cava filter was placed and patient was transferred to the intensive care unit. Following return from the cath lab, therapeutic hypothermia was instituted given persistent Glasgow coma scale of 8. A temperature of 33°C was targeted. This was maintained for 24 h, followed by a gradual rewarming (0.25°C/hour). In addition, he was anticoagulated with a heparin drip after local hemostasis was achieved at the bedside by the orthopedic surgeons. After 24 hours, the patient was rewarmed. He was successfully extubated 2 days later. He returned to neurologic baseline with normal cognition over the next several days. He was transitioned to Coumadin and was discharged home.

The decision to continue CPR beyond 20 minutes was based on the observed nature of the arrest, the availability of a large number of staff to sustain good quality CPR demonstrated by ETCO_2_ monitoring, and the persistence of a high BIS score suggesting persistent brain activity.

## 3. Review of Published Case Reports with Prolonged Resuscitation

### 3.1. Materials and Methods

We performed a PubMed search of “Prolonged Resuscitation” of all published articles from 1947 to 2013. 3,826 publications were found. Subsequent filters applied included the following: “Human Species”, “Case reports”, “Abstract”, and “English language”. 491 articles met the above criteria and were obtained by online access and through interlibrary loan. All 491 articles were reviewed by 2 pulmonary and critical care physicians. Articles were included in this study if they included a detailed description of the resuscitative effort during cardiac arrest (such as immediate cause of arrest, latency to chest compression, duration of chest compression, amount of defibrillation, and number of returns of spontaneous circulation), if such effort lasted at least 20 minutes and if there was a description of the outcome including death or neurological status.

### 3.2. Results

We identified 71 case reports published describing prolonged resuscitation after cardiac arrest. Some of the reports had more than 1 patient, and therefore a total of 82 patients are included in our review. All cases were published between 1980 and 2013 [7–77].

The baseline characteristics of the patients are listed in Table 1. The mean age was 43 ± 21 years. The main causes for the cardiac arrests were acute myocardial infarction (AMI) (29%), followed by hypothermia (21%) and pulmonary emboli (PE) (12%). Other etiologies include drug overdose (6%) and arrhythmia (7%). There were no cases of sepsis.

**Figure 1:**
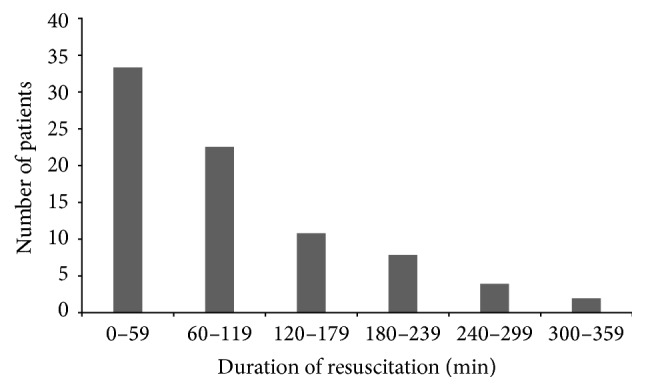
Duration of the cardiopulmonary resuscitation in the 82 patients.

The latency to CPR had an average duration of 2 ± 6 minutes (Table 2). The initial cardiac arrest rhythm was ventricular tachycardia or fibrillation in 41 cases (50%), and pulseless electrical activity or asystole in 37 cases (45%). The median duration of chest compression was 75 minutes with a range of 20 to 330 minutes (Table 2, Figure 1). Seven patients only had 20 to 30 minutes of resuscitation. Quality of chest compression was deemed good in 96% of the patients in these reports, even though the way this was determined is not clarified.

Several types of adjunct therapies were used (Table 3). Extracorporeal membrane oxygenation (ECMO) was used in 15 cases without the presence of a pulse, for a mean duration of 2.7 ± 3.9 days (range 3 minutes to 11 days). Thirteen patients received thrombolysis, including 9 out of the 10 patients with PE, as well as 3 patients with acute myocardial infarction. Rewarming was performed in 16 of the 17 hypothermic patients (94%). In 1 case of hypothermia, cardiac arrest occurred after starting the rewarming process, and the patient was kept hypothermic thereafter. Hypothermia after return of spontaneous circulation (ROSC) was reported in 13 patients, and cardiac pacing was done in 8 patients.

Post-ROSC complications (Table 4) were mainly respiratory (27%), secondary to pulmonary edema and bronchopneumonia. There was 1 case of hemothorax, 2 cases of pneumothorax, and 2 reported flail chest after chest compression. Renal failure occurred in 15 patients (18%), while liver hematoma was reported in 2 cases. There was 1 case of deformed aortic valve that was recently implanted.

Interestingly, signs of life during CPR were reported in 10 cases (12%). These signs were present without evidence of spontaneous circulation and included making respiratory efforts [15, 22, 30], sucking on the ET tube [11], hand movements [22–24, 48], opening eyes to call [22], tracking people around [22], occasional gaging and blinking [49], remaining conscious [22, 23], responding to name [39], and following simple commands [22, 39].

#### 3.2.1. Survival

Five patients (6%) did not recover after resuscitation. At 28 days, 13 patients were dead (16%). At 6 months, there was one additional death. No patient died between 6 and 12 months. The success rate of these resuscitation cases at 1 year was 68/82 (83%).

#### 3.2.2. Neurological Outcome

At 1 year, 50 patients (61%) had full neurological recovery, 14 patients (17%) had a good cerebral performance defined as a cerebral performance category (CPC) of one [78, 79], and 3 patients (4%) had moderate neurological deficit defined as CPC of 2. The neurological outcome was not reported in 1 patient. Overall 64/82 (78%) of the patients had a full neurological recovery or a good cerebral performance after prolonged resuscitation.

## 4. Discussion

Studies of the effect of the duration of resuscitation on clinical outcome are few. A recent retrospective analysis of out-of-hospital cardiac arrests showed a decrease in the probability of survival to hospital discharge with a good functional outcome with each minute of cardiopulmonary resuscitation. The probability of good functional outcome after 15 minutes of CPR was down to 2%, compared to 75% for patients resuscitated for 10–15 minutes [80]. In another study from Taiwan, the rate of ROSC was more than 90% in patients resuscitated for less than 10 minutes, compared to 50% when the CPR duration was more than 30 minutes [81].

Based on these studies, it appears that the need for longer resuscitation is associated with worse outcome. On the other hand, in a review by Goldberger, longer duration of resuscitation was associated with a higher survival rate, especially in patients with an initial rhythm of PEA or asystole [3]. Finally, an observational study by Cha et al. evaluated the duration of CPR and showed that resuscitation rate was 25.5% and survival rate was 5.6% when duration of CPR was more than 30 minutes [82]. The difference with our rate is most likely secondary to the publication bias inherent to the retrospective nature of our review.

Our reported and reviewed cases describe a unique group of patients that received cardiopulmonary resuscitation for a duration that far exceeded the average. In fact, the median CPR duration was 75 minutes, compared to an average of 17 minutes for in-hospital cardiac arrest [3] and 17.5 minutes for out-of-hospital cardiac arrest [80]. In addition, in 18% of the reviewed cases, the use of ECMO without a pulse was performed for a median duration of 55 minutes. This adds to the total duration of resuscitation. The 1-year survival was 83%, significantly higher than the overall survival rate to hospital discharge of 17% for in-hospital cardiac arrest [5] and of 5% for out-of-hospital cardiac arrest [6]. The neurological outcome was impressive as 78% of the patients achieving a full neurological recovery or CPC1.

A major drawback in our study is the presence of publication bias. Positive results' bias occurs when authors are more likely to submit, or editors accept, positive compared to negative or inconclusive results cases [83]. Cases of prolonged resuscitation with successful outcome are more likely to be written and accepted for publication compared to similar cases with poor outcome. On the other hand, conducting a randomized controlled trial may not be ethical. Despite that we believe that our study has identified a group of patients that can benefit from prolonged resuscitation. These patients were generally young, with no significant comorbidities. The cardiac arrest etiologies were generally reversible and correctable. Most arrests were witnessed, followed by immediate CPR, with advanced life support care, including ECMO when available. These represent the chain of survival factors advocated by the 2010 American Heart Association (AHA) guidelines, which include immediate recognition and activation, early CPR, rapid defibrillation, effective advanced life support, and integrated post-cardiac arrest care [84].

Physicians are frequently responsible for determining the maximal duration of resuscitation for each patient. We do not believe that an absolute duration of CPR is adequate for all patients. Rather, physicians should base the decision to continue CPR on several factors that affect the chances of survival after cardiac arrest. These factors include the patient baseline status, coexisting comorbidities, latency to CPR, latency to defibrillation, and adequacy of chest compression which should be monitored with ETCO_2_ and/or diastolic blood pressure [85].

Cerebral perfusion is the goal for CPR. BIS has been used to monitor the hypnotic state in the operating room and to titrate sedation in the ICU [86]. It has also been shown to reflect changes of cerebral perfusion during resuscitation of an animal model of shock [87]. BIS monitoring during CPR was used by some to assess cerebral perfusion as well as adequacy of chest compression [88]; some authors even suggested that a level greater than 30 to 40 during CPR is a reasonable target for better neurological outcome [89, 90].

Even though BIS can artificially be increased by movement artifact such as chest compression [91], providers should consider the need for sedatives in patients undergoing CPR who may exhibit signs of awareness. In our case, the patient had no such signs.

While there is no proven correlation between BIS monitoring and neurological outcome after CPR, our case report shows its potential benefit; we believe that a high level should be encouraging to the team to continue their effort as it reflects adequate perfusion; however a low level is more difficult to interpret.

The underlying disease causing the cardiac arrest can significantly affect the outcome of the resuscitation. Of all etiologies, four specific diagnoses constituted 67% of all reviewed cases. These are acute myocardial infarction, hypothermia, pulmonary emboli, and drug overdose. These etiologies should deserve special consideration by the treating physician for prolongation of CPR duration as well as other adjunctive therapies such as ECMO. In fact out-of-hospital cardiac arrests of cardiac origin have been associated with a better outcome compared to those of noncardiac origin [92]. In cases of cardiac arrest secondary to hypothermia, multiple case reports indicate survival after prolonged CPR and prolonged downtime. Based on these case reports the AHA recommends continuing resuscitative effort until rewarming has been provided [93]. In 10 of our 59 reviewed patients (17%), hypothermia was the cause of the arrest. Rewarming was provided in all these patients. Associated conditions included drowning in 1 case, diabetic ketoacidosis in 1 case, and drug overdose in 1 case. Only 1 death was reported in this population after 48 hours of admission. One study found that when cardiac arrest occurs secondary to PE, the diagnosis can be missed in 30% of the cases and thrombolytic therapy can significantly increase the rate of ROSC (81% versus 43%, *p* = 0.03) [94]. In our review, 7 patients (12%) were diagnosed with PE. All of them received thrombolytic therapy and survived to discharge with good neurological outcome. The initial rhythm was pulseless electrical activity (PEA) in 5 of the 7 patients (72%) and asystole in the other 2, consistent with the previously reported high rate of PEA (63%) and asystole (32%) in patient with PE [94].

ECMO may improve outcome after cardiac arrest when compared to standard CPR [95]. It may be particularly useful in cases of witnessed arrest, brief no-flow duration or when the underlying circulatory disease is amenable to immediate cardioversion. ECMO may also be beneficial as a bridge to correcting reversible conditions such as hypothermia and drug intoxications. Fifteen patients (25.4%) in our review were treated with ECMO. The primary cause of arrest was acute myocardial infarction (5/15), hypothermia (4/15), hyperkalemia (2/15), myocarditis (2/15), hypertrophic cardiomyopathy (1/15), and arrhythmia (1/15). There was only one reported death in this group. Fourteen patients were discharged home with good neurological function.

An interesting finding was the presence of signs of life in the absence of spontaneous pulse. These signs ranged from spontaneous respiratory effort to following commands while receiving chest compression. These signs disappeared when CPR was withheld to evaluate for ROSC. This could be explained by good CPR quality leading to a sufficient brain perfusion. In such cases, it is important to continue good resuscitative effort and minimize CPR interruption. Spontaneous pulse should only be checked at the recommended frequency to minimize brain injury from hypoperfusion.

## 5. Conclusion

Our review suggests that the decision to continue or stop CPR should not be based solely on the duration of resuscitation. Factors that affect the outcome include the patient's baseline condition, the reversibility of the cause of the arrest, the latency to starting CPR, the quality of CPR, and the availability and expertise in ECMO. It is possible to encounter signs of life during CPR which should be interpreted as evidence of good perfusion to an intact brain. It is important not to interpret these signs as evidence of ROSC and to minimize any interruption in chest compression.

## Figures and Tables

**Table 1:** Baseline characteristics of the 82 patients.

Baseline characteristics	*n* = 82 (%)
Age (years)	
Mean (SD)	43 ± 21
Median (range)	42 (0.1–88)
Gender, M/F	46/36
Location, *n* (%)	
Inpatient	40 (49)
Outpatient	42 (51)
Preexisting disorders, *n* (%)	
HTN	7 (9)
Diabetes mellitus	7 (9)
Cardiovascular disease	13 (16)
Chronic renal insufficiency	5 (6)
Malignancy	5 (6)
Cerebrovascular disease	1 (2)
Cause of the arrest, *n* (%)	
Acute myocardial infarction	24 (29)
Hypothermia	17 (21)
Pulmonary emboli	10 (12)
Arrhythmia	6 (7)
Drug overdose	5 (6)
Hyperkalemia	4 (5)
Myocarditis	3 (4)
Drowning	2 (2)
Diabetic ketoacidosis	2 (2)
Postelectroconvulsion therapy	1 (1)
Anaphylactic shock	1 (1)
Electric shock	1 (1)
Hemorrhagic shock	1 (1)
Drug induced paralysis	1 (1)
Abdominal aneurysm rupture	1 (1)
Cardiomyopathy	1 (1)
Amniotic fluid embolism	1 (1)
Lidocaine toxicity	1 (1)

**Table 2:** Characteristics of the cardiac arrests.

EKG rhythm at the beginning of CPR, *n* (%)	
Ventricular fibrillation	32 (39)
Ventricular tachycardia	9 (11)
Asystole	21 (26)
Pulseless electrical activity	16 (20)
Torsade de pointes	1 (1)
Latency to CPR (minutes)	
Mean (SD)	2.0 ± 6
Median (range)	0 (0–40)
Duration of (CPR) (minutes)	
Mean (SD)	97.5 ± 74.8
Median (range)	75 (20–330)
Quality of chest compression, *n* (%)^*∗*^	
Good	49/51 (96)
Interrupted	1/51 (2)
Mechanical chest compression	6/51 (12)
Defibrillation, *n*	
Mean (SD)	6.8 ± 13.5
Median (range)	3 (0–99)
Number of return of spontaneous circulation (ROSC)	
Mean (SD)	1.2 ± 0.6
Median (range)	1 (0–3)

^*∗*^Quality of chest compression reported in 51 patients only.

**Table 3:** Use of adjunct therapy.

ECMO	
Patients in whom ECMO was used without	15
a pulse, *n*	
Duration of ECMO without pulse	
Mean in minutes (SD)	3929 ± 5738
Median (range)	55 (3 minutes–11 days)
Total duration of ECMO usage (days)	
Mean (SD)	4.5 ± 3.9
Median (range)	3.5 (1–11)
Thrombolysis, *n* (%)	13 (15.8)
Thrombolysis for pulmonary emboli	9
Thrombolysis for acute myocardial infarction	3
Thrombolysis for refractory arrhythmia	1
Stent placement, *n* (%)	9 (11)
Stent placement during cardiac arrest	1
Stent placement after ROSC	8
Rewarming in the 17 patients with hypothermia, *n*	16
Hypothermia after ROSC, *n*	13
Open cardiac massage, *n* (%)	3 (3.6)
Cardiac pacing, *n* (%)	8 (9.7)
Amputation of ischemic leg, *n* (%)	1 (1.2)

ECMO: extracorporeal membrane oxygenation.

ROSC: return of spontaneous circulation.

**Table 4:** Prolonged CPR related complications after prolonged resuscitation.

Respiratory^*∗*^, *n*	22
Pulmonary edema	15
Pneumonia	7
Pneumothorax	2
Hemothorax	1
Rib fracture	4
Renal failure, *n*	15
Low ejection fraction, *n*	7
Neurological, *n*	
Intracranial hemorrhage	3
Ischemic stroke	1
Seizure	5
Bleeding disorders, *n*	4
Rhabdomyolysis, *n*	4
Liver hematoma, *n*	2
Deformation of aortic valve, *n*	1
Colon ischemia, *n*	1

^*∗*^Some patients had more than one complication.
